# The Myrtiformis Muscle: Identification of a Forgotten Entity That Is Distinct From the Depressor Septi Nasi Muscle

**DOI:** 10.7759/cureus.36214

**Published:** 2023-03-16

**Authors:** Mehmet E Yeği̇n, Okan Bilge, Servet Çelik

**Affiliations:** 1 Plastic, Reconstructive and Aesthetic Surgery, Istinye University Hospital, Istanbul, TUR; 2 Anatomy, Ege University Faculty of Medicine, Izmir, TUR

**Keywords:** rhinoplasty, nose, facial muscle, cadaver, anatomy

## Abstract

Introduction: Nasal musculature anatomy is a topic that plastic surgeons pay attention to. However, the presence and role of the myrtiformis muscle (MM) remain controversial. To elucidate these aspects, an anatomy-based study was conducted.

Materials and methods: Seven midsagittally split and two total cadaver head's nasal bases, embalmed with modified Larssen solution (MLS), were dissected for MM anatomy. The features of this muscle were photographed, and a video of its function was recorded.

Results: It was found that MM originates from the maxillary alveolar process and continues as two heads, one reaching the alar base with spicular fibrotendinous endings and the other extending to depressor septi nasi fibers. Owing to its bi-vectoral muscle fibers, MM is found to constrict the nares by simultaneously forcing the alar base and lowering the columella. It was also found that left-sided muscles were larger than right-sided muscles.

Conclusions: The MM is found to be a constrictor muscle of the nares in this study, contrary to recent observations.

## Introduction

The anatomy of the nose is an exciting topic in plastic surgery. In most cases, approaches and techniques describing a better outcome with rhinoplasty only change the anatomical relationships between cartilaginous, bony, muscle, and skin-related structures, such as onlay tip grafts, spreader flaps, or osteotomies [1]. Thus, the anatomical knowledge of this region is essential for surgeons. Anatomical relationships have also been observed in cadaveric studies. Cadaveric studies have been conducted on the widespread use of formaldehyde (FA). However, these studies fall short of providing surgeons the tissue quality and sense, as FA hinders close-to-real estimation of muscle functions, reflections on the skin, or dissection quality because of the effects of FA on tissue and the hazards of FA [2]. Therefore, fresh-frozen or live-like embalmed cadavers have been used. As an example of these “live-like” embalming techniques, cadavers embalmed in modified Larssen solution (MLS) were used in this study [3].

Myrtiformis muscle (MM) is a fan-shaped muscle that begins from the myrtiform fossa at the anterior surface of the alveolar process of the maxilla [4]. First mentioned in Sappey’s work in 1876, the MM is referred to as a depressor muscle of the nasal septum [5]. In addition, it is thought to function as a dilator of the nares and cause loss of tip rotation [5]. However, to our knowledge, these functional properties are not shown in an anatomical study. Therefore, our study primarily aimed to clarify the existence and functions of this muscle.

## Materials and methods

This study was conducted in the anatomy department, and all cadavers were obtained according to the country's relevant laws. This study has been approved by the Ege University Ethics Committee of Clinical Studies (approval no. 23-3T/23). A total of 19 cadaver hemi-faces (left or right sides of the nasal base) were dissected for this study. At the beginning of the study, seven 10% FA-fixed cadaver heads and one sagittal half (eight hemi-faces in total) were dissected to determine the topographic anatomy. After an anatomical orientation to the region, 11 hemi-faces of MLS-embalmed cadavers (two heads and seven sagittal halves) were examined to observe the MM’s position, dimensions, and function.

Muscle measurements were performed using the Adobe Photoshop 2023 (Adobe Inc., Mountain View, CA, USA) software program. Therefore, a millimetric transparent scale was placed on each plane during the photography. Eleven MM in nine cadaver heads were observed. The origin, insertion, and dimensions of the muscles in millimeters were identified.

Dissection procedure

**Figure 1:**
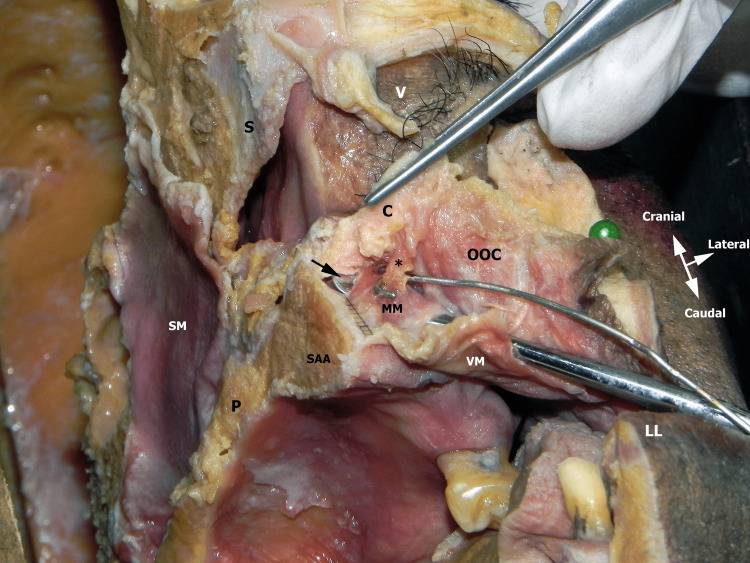
The left half of a cadaver head with a right-inferior oblique view. The green pin was used to retract the OOC and skin laterally. Observed is the tip of the periosteal elevator (black arrow) that has lifted the body of the MM right after its periosteal insertion. The wire hook strains the fibers of MM (*) that reach the columellar base (C) by joining the DSN muscle. The body of the MM (over the periosteal elevator) continues toward the base of the left nasal ala. P: Hard palate, SAA: Superior alveolar arch, VM: Vestibular mucosa, SM: Nasal mucosa of septum, LL: Lower lip, V: Left nasal vestibulum, OOC: Orbicularis oris complex, MM: Myrtiformis muscle, DSN: Depressor septi nasi

In all the cadaver heads, first, the vermilion was incised transversely. A medial approach was also applied to half of the heads. After the vermilion incision, the orbicularis oris muscle (OOM) was identified, and dissection was performed in the supramuscular plane to identify its parts. Cranial dissection was performed to determine the muscle belly and attachments of the depressor septi nasi (DSN) muscle. In the area at the beginning of the DSN, called the nasal modiolus, a midline incision was made to discard the superficial layer consisting of OOM-DSN. Subsequently, submuscular dissection was performed. Precise dissection was performed to avoid cutting or snatching the muscle fibers through deeper planes. Dissection was performed until the anterior nasal spine was reached. Only the mucosal and supraperiosteal layers remained, and the muscle fibers reached the superficial planes after this phase (Figure 1).

**Figure 2:**
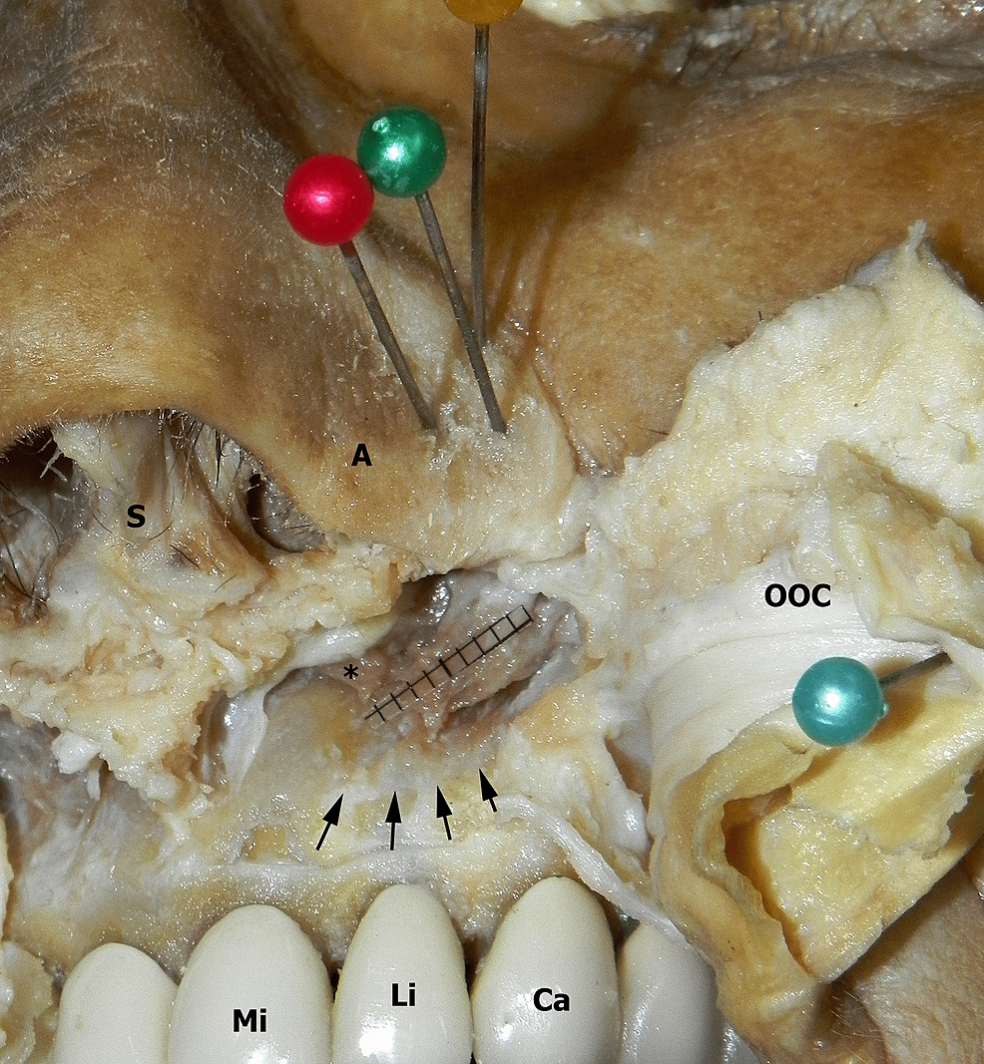
Antero-inferior view of the left nasal base. The blue pin retracts the folded mucosa and OOC. The red, green, and yellow pins are inserted vertically into the LLC base. Trajectories of the pins show the fibrotendinous endings of the MM. These fibers reach the base of the LLC. On the lower part of the figure, the MM body with a transparent millimetric scale sheet can be seen. Black arrows indicate the insertion line of detached MM. *: fibers to the superficial layer of orbicularis oris-DSN fusion, DSN: Depressor septi nasi, A: Nasal ala, S: Nasal septum, Mi: Median incisor teeth, Li: Lateral incisor teeth, Ca: Canine teeth, OOC: Orbicularis oris complex, MM: Myrtiformis muscle, LLC: Lower lateral cartilage

The dissection was extended to the periosteal plane. When advanced to the origin of the MM, the MM and its fibers departing from the muscle belly were delicately dissected until they were inserted into the subcartilaginous fibro-adipose connective tissue and periosteum under 2.5x magnification. Lastly, the subperiosteal plane was opened to identify the origin of the MM (Figure 2).

**Figure 3:**
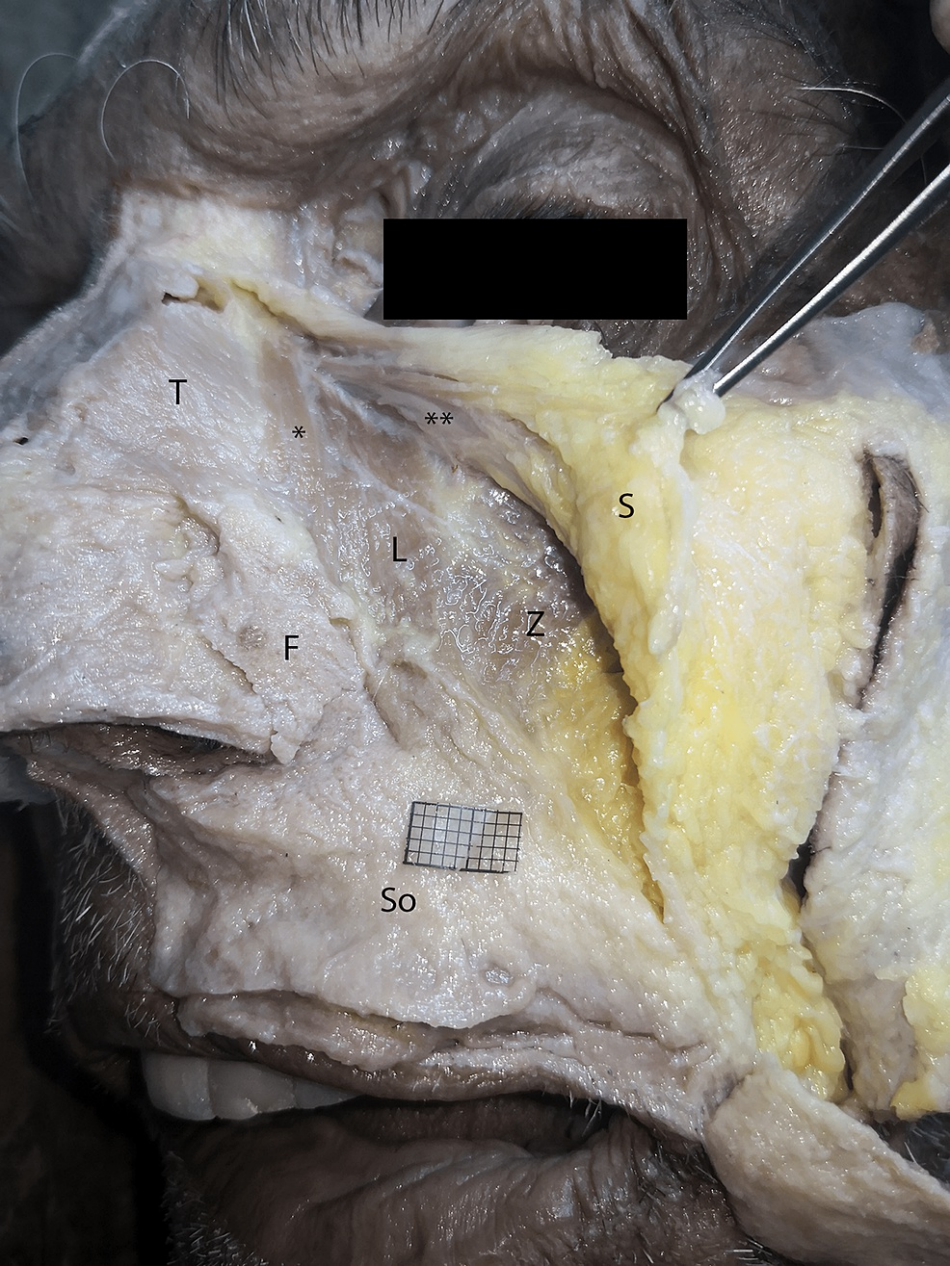
Superficial plane of the lateral dissections. The LLSAN (*), zygomaticus minor (Z), and transverse part of the nasal (T) muscles can be seen on the same plane under laterally deviated SMAS (S). SMAS: Superficial musculoaponeurotic system, So: SMAS over the orbicularis oris muscle, F: fibrolamellar tissue of the nasal wing, **: orbital fibers of orbicularis oculi, LLSAN: Levator labii superioris alaeque nasi

Lateral dissection was performed to widen the exposure and identify the surrounding soft tissue. The levator labii superioris alaeque nasi muscle (LLSAN) and transverse nasalis muscle (TNM) were identified in the same plane as the OOM (Figure 3).

**Figure 4:**
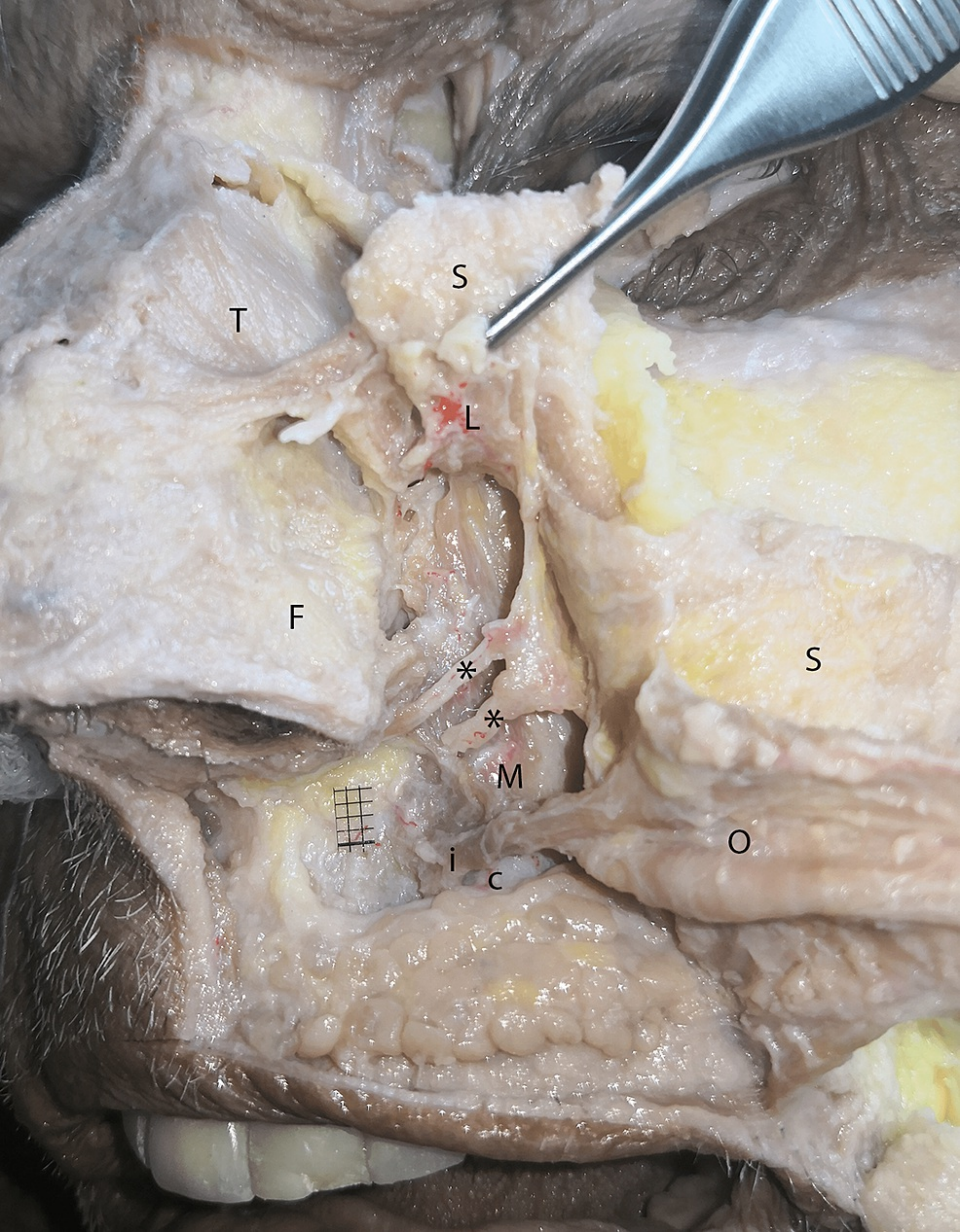
Second plane of dissection. The orbicularis oris (O), and LLSAN (L) muscles were detached and deviated laterally. The ILS (i), a part of the OOM, originates medially to the canine teeth juga (C). It is clearly demonstrated that the branches of the infraorbital nerve and vessels (*) are placed between the superficially placed LLSAN, zygomaticus minor, and OOM and the deeply placed MM. LLSAN: Levator labii superioris alaeque nasi, ILS: Incisivus labii superioris, OOM: Orbicularis oris muscle, MM: Mirtyformis muscle

On deepening the medial side of the dissection, underlying infraorbital neurovascular branches were encountered under the OOM and LLSAN (Figure 4).

**Figure 5:**
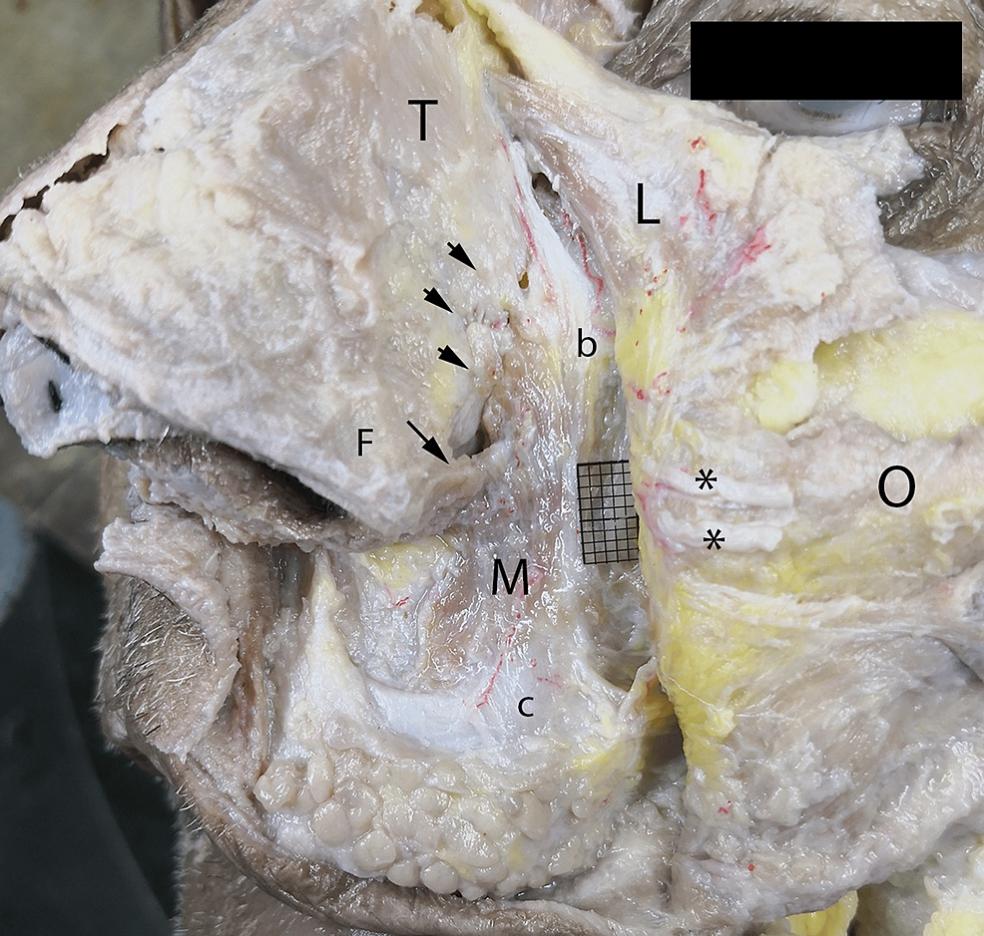
The deepest plane of dissection. The orbicularis oris (O), LLSAN muscles (L), and branches of infraorbital nerves (*) have deviated laterally. Medial fibers of the MM (M) curve acutely to insert the fibroareolar tissue (F) of the nasal wing (indicated by the long arrow). Lateral fibers are longer and slope medially to insert the fibrous tissue covering the LLC and accessory cartilages (indicated with short arrows). A lateral fibromuscular tissue bundle forms a border-like structure for MM (b). C: The tip of the juga alveoli of the canine teeth, T: transverse part of nasalis muscle, MM: Myrtiformis muscle, LLSAN: Levator labii superioris alaeque nasi, LLC: Lower lateral cartilage

When infraorbital neurovascular structures, LLSAN and OOM were retracted laterally, MM was observed. With a clear distinction of the muscle from TNM, the end fibers of the muscle have different angles, traversing the lower lateral cartilage (LLC) base (Figure 5).

In addition, the MLS-fixation method left muscle fibers completely intact, and it was possible to observe muscle function. Thus, when the muscle was dissected, its contraction was simulated to observe functional effects on the nasal structures. The contraction simulation was performed by pulling the muscle belly while observing the insertion area.

## Results

**Figure 6:**
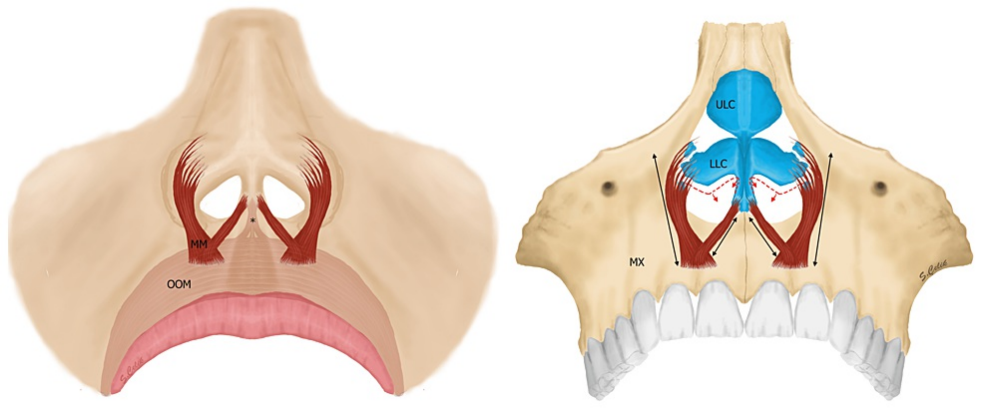
An anterior view illustration of the location and action of the MM Left: MM is in the deeper plane than the DSN (*) and OOM. The DSN and OOM are depicted as faded. Right: Two-headed arrows pointing out the vectors of muscle forces. Dotted lines show the new position of LLC and medial crura when the muscles are contracted bilaterally. MM: Myrtiformis muscle, DSN: Depressor septi nasi, OOM: Orbicularis oris muscle, LLC: Lower lateral cartilage, ULC: Upper lateral cartilage, MX: Maxilla Illustration created by author Servet Celik

The muscle begins at the periosteum of the myrtiform fossa at the maxillary alveolar process. In most cases, the origin of MM was found between the alveolar yokes of the upper central and lateral incisors (as seen above in Figure 2). In some cases, the line expanded laterally to the medial side of the alveolar yoke of canine teeth. Insertion was found not only in one area, but the muscle fibers ended up with tendon-like attachments scattered to the fibro-adipose tissue right under the base of the nasal LLCs (Figure 5). Beginning relatively medially, its course through the base of the LLCs gives an inferomedial vector of force. Additionally, as mentioned above, some fibers from this muscle reached the medial crus by joining a more superficial layer of the orbicularis oris-DSN fusion (as seen above in Figure 2, and shown below in Figure 6). 

The action of MM included two vectors. The first is an inferomedial vector, as mentioned above. Its contraction causes inferomedial displacement of the LLC and the lateral alar base. In addition, the fibers found to be bridged between the MM and DSN cause septal depression. When these vectors are combined, MM causes narrowing of the nostril, with inferomedial displacement of the LLC and inferior displacement of the septum nasi, while over-rotating the nasal tip (as illustrated above in Figure 6). 

The MM was straight on the periosteum. The fascia surrounding the muscle belly separates the muscle from more superficial layers such as the orbicularis oris complex (OOC). The only point at which the fascia loses its integrity is at the fibers, leaving the muscle belly to join the orbicularis oris-DSN fusion. Otherwise, the fascia continues cranially to join the insertions of muscle fibers. 

The left-sided muscular dimensions were slightly bigger than the right-sided ones. The muscle originated from a 2 cm × 2 cm base with an average of 3,96±0.32 cm2 on the alveolar process. The muscle body lengths were between 4.1 cm to 4.6 cm, with an average of 4.34±0.17 cm. The area where spicular extensions begin to reach LLCs was measured as 0.6 to 1.21 cm2, with an average of 0.96±0.13 cm2 (Table 1).

**Table 1:** Calculated area and dimensions of the dissected MM MM: Myrtiformis muscle, SD: Standard deviation, R: Right side, L: Left side

Cadaver #	Dimensions of origin (cm^2^)	Length of the muscle (cm)	Dimensions of insertion (cm^2^)
1L	4.18	4.4	0.84
1R	3.80	4.5	0.96
2L	4.60	4.2	0.99
2R	3.99	4.1	1.04
3L	4.32	4.6	0.77
3R	3.78	4.5	0.80
4L	3.58	4.3	1.21
4R	3.57	4.1	1.08
5L	4.18	4.4	1.08
5R	3.78	4.4	0.88
6L	3.78	4.2	0.96
Average dimensions & SDs	3.96 (±0.32)	4.34 (±0.17)	0.96 (±0.13)

We observed that each case had 15 to 20 spicular fibrous bands attached to the LLCs.

## Discussion

Rhinoplasty results are affected by regional musculature, skin properties, and external impacts [6-8]. In addition, modifications of the nasal musculature are effective against various deformities [6,9-11]. Hence, nasal musculature must be considered an effector of rhinoplasty outcome.

There is a controversy over nasal musculature. There are many papers about nasal musculature and its manipulation in rhinoplasty [4-6]. However, the nasal base musculature remains unclear. The DSN is the main target of these studies [4-7].

Depressor septi nasi is shown to be a muscle beginning from the nasal modiolus and reaching the OOC lying on the same plane as the OOM. Moina et al. illustrated the anatomy and derivation of DSN. This illustration showed a muscle beginning from the caudal tip of the septum and reaching the OOM clearly [12]. Furthermore, it is shown to be the inferomedial border of the “deep pyriform space.” In this study, the depressor muscle was shown to lie immediately under the nostril base [13]. However, our dissections showed a deeper muscle that lies more profound in this space. When its function is considered, the DSN is blamed for depressing the tip, resulting in a drooping nose after rhinoplasty. Cutting this muscle may improve postoperative nasal projection [6,14]. In addition, other external nasal muscles contribute to external nasal valve collapse [15]. In some studies, these muscles aim to strengthen, resulting in better outcomes [16].

It was shown that a 4 cm long, two-headed muscle originating from the alveolar process of the maxilla in this study. Its origin has been previously described [8]. One of the muscle heads reached the nasal modiolus and DSN. The other head was attached to the LLC base with fibrotendinous endings lying medial to the nasolabial sulcus. With an inferomedial oblique vector, its contraction is supposed to pull the columellar base to the inferior base because of its attachment to the nasal modiolus while pulling the alar base inferomedially. In the posteroanterior plane, the vector becomes an anteroinferior oblique orientation, causing anteroinferior displacement of both structures.

In the middle of the 19th century, MM was already described and additionally named as “depressor labii superiors et alae nasi” [14]. After then, only the fossa maxillaris was named “myrtiform.” Eventually, in Terminalogia Anatomica, the MM does not exist, while the DSN is listed under the facial muscles. We believe these two muscles exist separately, and the nomenclature may be revised for this [17].

Subsequently, Hoeybergs et al. described an alar depressor muscle. It was mentioned as a “lost muscle” because it is not drawn in modern anatomy atlases. Their study indicated that this muscle begins from the canine fossa and reaches the alar base [18]. Their description in this study fits precisely with the description above. In addition, our dissections showed no signs of any other muscular structures adhering to this area. It was named MM, a nasal base muscle described by Delaire et al. in 1977 [9]. It has two parts: medial and lateral portions [8]. As mentioned above, this information supports our finding that the fiber branch reaches the modiolus. This also indicates a similar vector of forces to that of the DSN. Thus, we believe the medial portion similarly affects the DSN on the columellar base. The MM depresses the nasal tip, causing external valvular widening after transection in Le Fort I osteotomies [19]. This study showed the same predictable results, including narrowing the outer valve. However, Figallo et al. described this muscle as an expander of the nostrils [4]. In addition, Daniel et al. concluded that MM is a nostril expander, following Figallo et al. [4-5]. In contrast, our findings suggest that when its vectors are considered, contraction of this muscle does not widen the nasal rim but rather narrows it. With its branch to the superficial layer, this muscle can pull the septal base inferiorly, causing the same function as the DSN.

Another study, showing a muscular branch that is supposed to be the MM as a lateral branch of the DSN, also indicated that it narrows the nares [20]. The MM was not mentioned in another recent study, and fibers beginning in this area were advocated to be the beginning of the nasalis muscle [21]. Inherently, when we simulated its contraction by pulling the muscle's origin in the same direction as its vector, we observed the inferomedial bearing of the lateral nasal base, with the inferior movement of the modiolus, pulling the columella downwards. This causes the narrowing of the vestibule. This caused the nares to lose their width. Eventually, it increases the tip rotation while decreasing the projection and narrowing the external valve.

It is essential to mention the features of the cadavers used. Dissection of FA-fixed cadavers showed difficulties in determining the clean borders of the muscles. Dissection was made as delicate as possible, but a clear distinction between the muscular structures could not be made quickly. In addition, tearing of the muscle bellies was the most limiting factor in functional analysis. Additionally, tissue shrinkage occurs widely in FA [3,22]. Therefore, cadavers with FA were not included in this analysis. The cadavers used in our study, as introduced by Bilge (&) Celik, were fixed with MLS for simulation purposes. They showed that surgical courses with these cadavers offer a better and more live-like experience with dissection [3]. Furthermore, dissections in our study showed that these cadaver tissues were not dehydrated, and muscles had more tissue caliber and tensile strength than FA-fixed or fresh-frozen cadavers. Therefore, we performed the dissections easily and mimicked the contraction of the muscle without ripping it.

Left-sided muscles were larger than right-sided muscles, and measurements supported this observation. However, with the small cadaver numbers in this study, we could not statistically analyze the differences. In addition, histological workup could contribute to the precision of our research. Another limiting factor of our study was the need for electromyographic (EMG) evidence. In future studies, in vitro EMG in humans may reveal the function of this muscle with greater precision.

## Conclusions

The origin of MM, its insertion, and its functions have yet to be clarified. Our study shows that the MM is a separate muscle deeply situated in the orbicularis oris-DSN complex. It can be described as an effector of the nasal base anatomy, making it a possible future target of rhinoplasty procedures. As we could imitate its contraction, owing to the feature of the embalming solution, we determined the narrowing effect on the nostrils while functioning with the DSN muscle via the insertion of fibers into the septal base.
